# Epidemiology, etiopathogenesis, immune response, diagnosis, and complications of acute pancreatitis: current insights

**DOI:** 10.1080/07853890.2025.2580131

**Published:** 2025-11-09

**Authors:** Jakub Kosidło, Justyna Dorf, Joanna Kamińska, Olga Koper-Lenkiewicz, Joanna Matowicka-Karna

**Affiliations:** Department of Clinical Laboratory Diagnostics Medical University of Bialystok, Białystok, Poland

**Keywords:** Acute pancreatitis (AP), biomarkers, etiopathogenesis, inflammation, leukocyte, reactive oxygen species

## Abstract

The pancreas is a vital organ that performs exocrine and endocrine functions essential for digestion and glucose homeostasis. Acute pancreatitis (AP) is an inflammatory disorder with a rising global incidence, particularly in countries with a low socio-demographic index (SDI). It is characterized by abdominal pain, elevated pancreatic enzymes, and imaging-confirmed pancreatic injury. The disorder is commonly linked to gallstones, alcohol consumption, and metabolic disturbances. The pathophysiology of AP involves complex interactions between oxidative stress, intracellular calcium dysregulation, and immune cell activation. Reactive oxygen and nitrogen species contribute to acinar cell injury by driving apoptosis or necrosis and amplifying inflammatory cascades. Leukocytes, including neutrophils, monocytes, macrophages, lymphocytes, and platelets, then infiltrate pancreatic tissue and produce cytokines and chemokines, thereby exacerbating tissue damage. Pancreatic stellate cells play a central role in fibrosis by modulating the deposition of the extracellular matrix and promoting persistent inflammation. AP can lead to complications such as necrosis, bacterial superinfection, pseudocyst formation, and chronic fibrosis. The long-term risk of pancreatic neoplasia is enhanced by repeated inflammatory insults and oncogenic mutations. Early prediction of severe AP is critical. Clinical tools include biochemical markers, hematological indices, scoring systems, multimarker panels, and deep learning applications. This review integrates current knowledge on oxidative stress, immune responses, and tissue remodeling in AP. It highlights the underlying mechanisms of disease progression and the potential of biomarkers and predictive models to optimize clinical management.

## Graphical Abstract [1–5]

**Figure:**
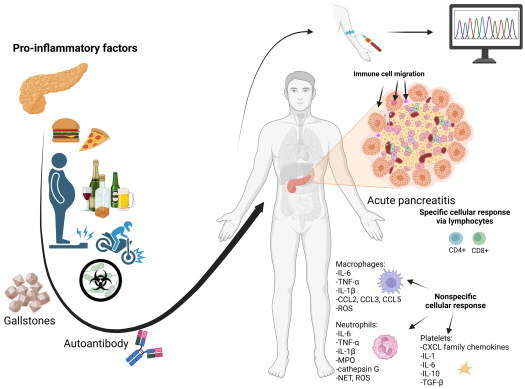

## 1. Introduction

**Figure 1.**
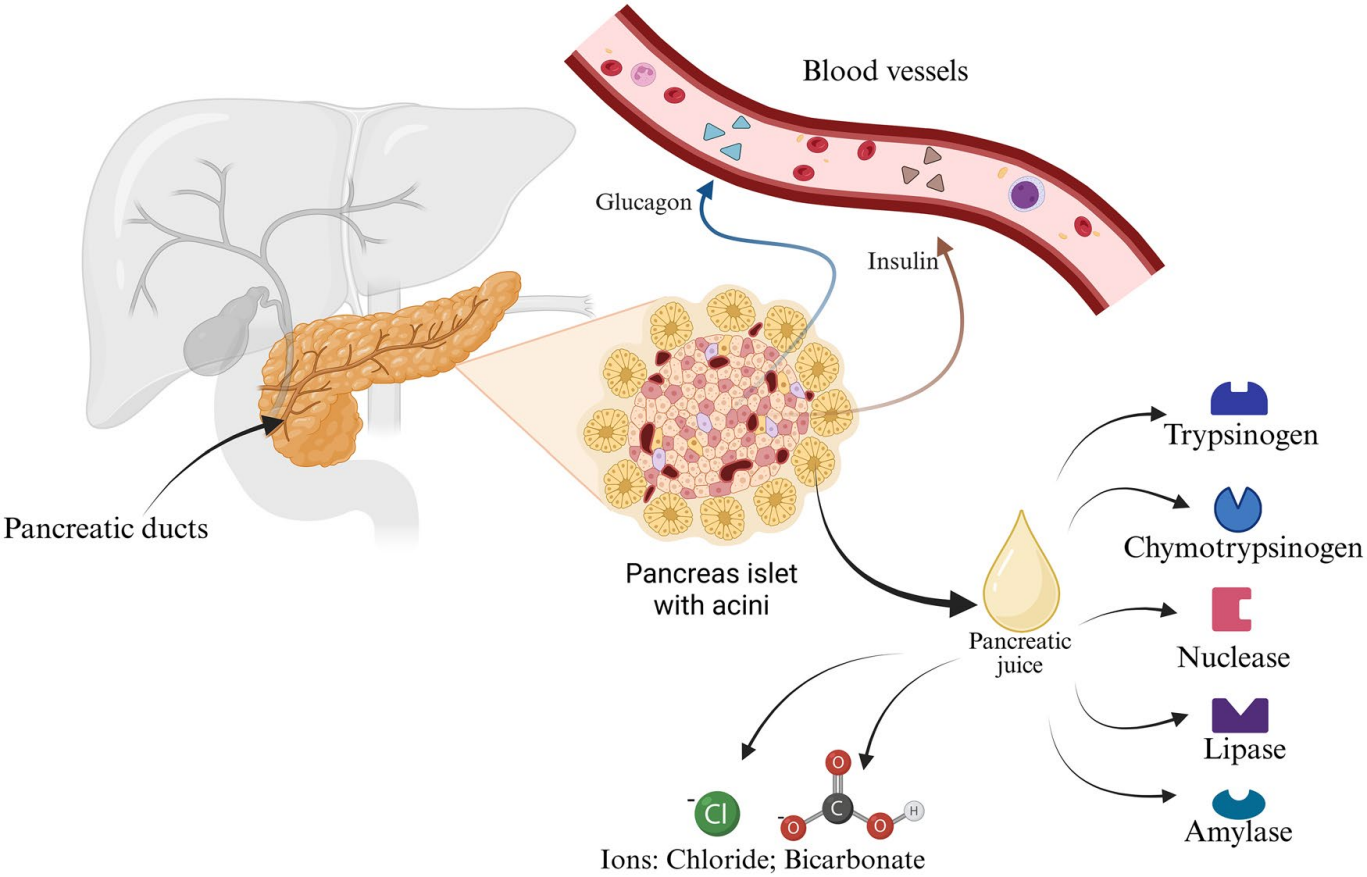
Anatomy and basic physiological functions of the pancreas [1,6–8].

The physiological glandular function of the pancreas is endocrine secretion [1–5]. It is evident that hormones regulating glucose metabolism, including insulin and glucagon, are released into the bloodstream [3]. The exocrine functions comprise the secretion of zymogens suspended in pancreatic juice into the duodenum [3]. These include proteases, lipases, and nucleases, which are synthesised in an inactive form (zymogens) in acinar cells [6,7]. Physiologically, they are transported as a liquid, colourless secretion through small ducts to larger vessels. Physiologically, they are transported as a liquid, colourless secretion through small ducts to larger vessels. The resulting fluid also contains chloride and bicarbonate ions, which buffer the pH of the pancreatic juice (Figure 1). It then flows into the pancreatic duct, from where it reaches the common outlet with the bile ducts at the duodenal papilla. Partially digested gastric contents, acidified by stomach acid and pepsin, enter this part of the intestine. The low pH, presence of pepsin and enterokinase activate trypsinogen and chymotrypsinogen to their proteolytic enzyme functions [3,7].

According to the 2012 Atlanta Classification, acute pancreatitis is characterised by the premature activation of pancreatic enzymes, which results in inflammatory and necrotic tissue changes [6]. The development of a severe inflammatory response may be promoted by oxidative stress [8,9]. Disrupting the redox balance leads to the accumulation of reactive oxygen species (ROS). These cause the oxidation of membrane phospholipids, integrins, cytoskeletal proteins, receptors and ion transporters [10]. The presence of activated proteolytic enzymes in pancreatic vesicles and ducts enables the degradation of proteins, including surface proteins, thereby disrupting cellular homeostasis. Additionally, changes in cell membrane potential and loss of elasticity lead to the release of DAMP (Damage-Associated Molecular Patterns) [6,10]. As cells break down, histones, heat shock proteins (HSP), High Mobility Group 1 Protein (HMGB1), ATP, nuclear DNA, mtDNA, and RNA are released [10]. Aseptic inflammation is recognised as the earliest manifestation of injury in AP. DAMP-driven sterile inflammation has been identified as a pivotal event underlying subsequent pancreatic injury, distant organ damage, and ultimately disease resolution. This highlights the central role of DAMPs in both the initiation and progression of acute pancreatitis (AP) [11].

Li et al. demonstrate an increase in the number of AP cases from 1,727,789 in 1990 to 2,814,972 in 2019. The increase in cases is predominantly observed in countries exhibiting a low socio-demographic index (SDI). It is noteworthy that in countries with a high SDI, a 7.9% decrease in incidence was observed during the studied period. A significant number of cases of acute pancreatitis (AP) have been observed in North American countries (257,777.8). However, from 1990 to 2019, the standardized incidence rate decreased by 16.6%. In contrast, in Eastern European countries, an 11.7% increase was noted during the same period, from 182,577 to 221,945 [12]. The World Health Organization (WHO) has reported a consistent increase in the number of deaths among elderly patients suffering from pancreatitis [13]. Marta et al. revealed a ninefold increase in the risk of mortality from acute pancreatitis in patients aged 70 in comparison to patients aged 20 [14].

The hospitalization of patients suffering from pancreatitis is generally multi-stage and complex. The treatment of acute pancreatitis (AP) is primarily focused on the utilisation of pharmacotherapy, dietary modifications, and the elimination of the underlying etiological cause, most often achieved through surgical intervention [13,15]. Imaging studies, endoscopic ultrasonography or duodenal intubation are required for diagnosis [13,15]. A delay in the diagnosis of acute pancreatitis (AP) can result in the onset of a severe form of inflammation, which increases the risk of complications such as multiple organ failure or death (Table 1) [16–18]. Teshima et al. analysed data on the direct cost of treating pancreatitis for Canadian patients in 2004–2005, which totalled 118 million dollars. In contrast, the cost in the USA exceeded 3.7 billion dollars [15]. Wadhwa et al. indicated in their study that the duration of hospitalisation for patients with pancreatitis in the USA was reduced from 6.3 days to 4.7 days [4]. However, as researchers point out, the cost of hospitalisation during the studied period increased by 118.6% [4].

**Table 1.** Frequency of deaths due to pancreatitis by age in selected countries [13].

Age groups	15–24			25–34			35–54			55–74			75+		
sex	M:	F:	All:	M:	F:	All:	M:	F:	All:	M:	F:	All:	M:	F:	All:
Lithuania	0.7	0	0.4	4.3	1.1	2.8	12.7	4.1	8.3	16.7	6.8	11.1	29.1	26.7	27.4
Estonia	0	0	0	3.1	0	1.6	6.0	0.6	3.4	11.0	4.5	7.4	28.0	7.4	13.5
Poland	0.6	0.2	0.4	3.5	0.5	2.0	6.8	1.4	4.1	11.0	11.0	4.6	22.2	18.2	19.7
Czechia	0	0.2	0.1	1.4	0.2	0.8	4.2	1.1	2.6	9.3	3.4	6.2	16.5	14.2	15.0
Denmark	0	0	0	0.3	0	0.1	2.1	0.4	1.3	7.1	3.1	5.1	19.8	16.4	17.8
USA	0.2	0.1	0.1	0.8	0.3	0.6	1.6	0.7	1.2	3.2	1.6	2.4	7.0	6.6	6.8
Cuba	0	0	0	0.4	0.1	0.3	1.8	0.7	1.2	4.4	3.5	4.0	9.9	7.0	8.3
Canada	0.2	0	0.1	0.3	0.2	0.2	0.8	0.4	0.6	2.3	1.2	1.8	9.7	9.8	9.8
Brazil	0.3	0.2	0.3	1.1	0.4	0.8	3.1	1.2	2.1	5.9	3.3	4.5	15.1	14.3	14.6
Colombia	0.1	0.1	0.1	0.3	0.1	0.2	0.8	0.4	0.6	2.9	2.1	2.4	9.8	8.1	8.8
Mexico	0.3	0.3	0.3	1.6	0.8	1.2	2.9	1.2	2.0	5.7	3.7	4.6	13.0	12.0	12.4
RPA	0.2	0.2	0.2	1.0	0.2	0.6	2.3	0.7	1.5	4.3	1.7	2.9	4.4	3.5	3.8
China	0	0	0	0.2	0	0.1	0.2	0	0.1	1.5	0.9	1.2	8.0	12.4	10.0
Kazakhstan	0.2	0.2	0.2	2.6	0.7	1.6	11.5	2.5	6.9	23.8	10.4	16.1	52.6	57.7	56.2
UAE	0.4	0.2	0.4	0.2	0	0.1	0.2	0	0.2	0	1.9	0.5	4.5	10.0	6.2

Legend to Table I: F, female; M, male.

This review aims to provide a comprehensive overview of the pathophysiology, clinical presentation, and complications of acute pancreatitis. Particular emphasis is placed on the roles of oxidative stress, inflammatory and immune cell responses, and the mechanisms that lead to fibrosis and neoplastic transformation. Additionally, this review summarizes the current knowledge of biochemical markers and clinical scoring systems that are used to predict the severity of AP.

## 2. Search criteria

A comprehensive literature search was conducted in open-access databases, primarily PubMed, using the following keywords: “acute pancreatitis” AND “etiology” AND “pathogenesis” AND “nonspecific cellular response” AND “diagnostic parameters” AND “diagnostic preventive parameters” AND “diagnostic preventive markers” AND “reactive oxygen species” AND “ROS” AND “pancreatic stellate cells”.

The inclusion criteria involved publications identified by the aforementioned search terms, including meta-analyses, review articles, and original research studies. Studies published between 2010 and 2025 were given preference, unless they were historically relevant papers of fundamental importance. Only articles written in English and available in full-text format were included.

The exclusion criteria included studies that exclusively focused on chronic pancreatitis, case reports, and publications that did not have full-text accessibility.

### 2.1. Symptoms and criteria for diagnosing acute pancreatitis

The primary criterion for diagnosing AP is the occurrence of diffuse pain in the epigastrium. Patients frequently present with fever and non-specific symptoms, such as nausea and vomiting. In severe cases of the disease, painful and tender epigastric swelling is observed, in addition to the occurrence of shock symptoms [6]. According to the Atlanta Consensus, the presence of AP is determined by the occurrence of two out of three main criteria. The occurrence of pancreatic-type pain, at least threefold increase in serum pancreatic enzyme activity (i.e. lipase and amylase), and the manifestation of changes characteristic of AP in imaging studies (i.e. contrast-enhanced computed tomography (CECT); magnetic resonance imaging (MRI); ultrasound (USG)) have also been observed [6].

### 2.2. Factors contributing to the occurrence of AP

**Figure 2.**
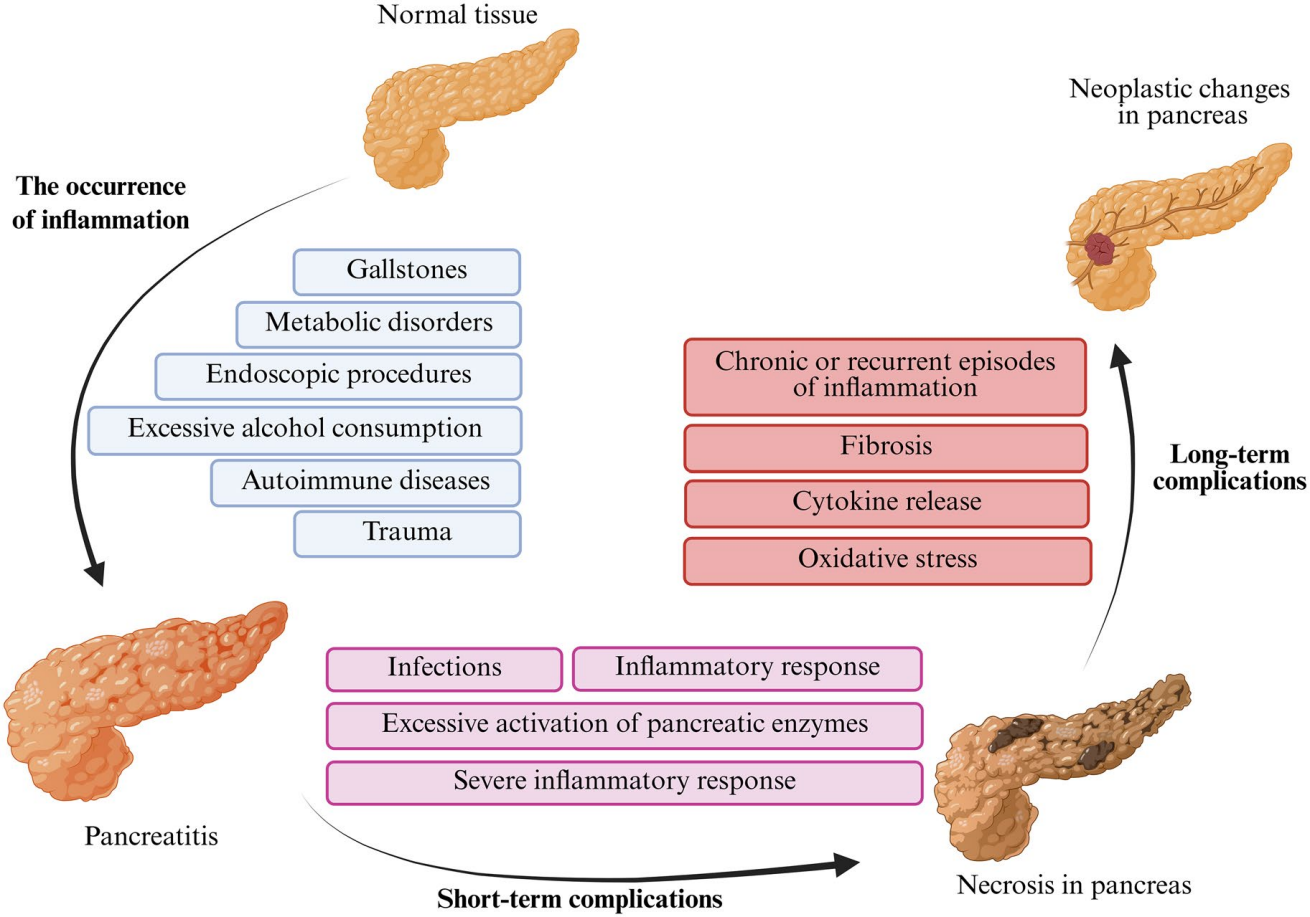
Etiopathogenesis of pancreatitis and complications [7,13–15,21,96,97,99–102].

Studies suggest that gallstones, excessive alcohol consumption, and hypertriglyceridemia are the most common contributors to the occurrence of AP (Figure 2). These main etiological factors of AP are often interrelated and can interact with each other [19]. It is observed that the development of gallstones is favored by dietary errors and irregular food intake. The occurrence of these symptoms may also be attributed to reduced fluid intake or disease states that result in hypercalcemia, such as hyperparathyroidism. Excessive alcohol consumption can also have multifactorial influences on the development of AP [19–21]. Ethanol has been demonstrated to inhibit the antidiuretic effect. The presence of vasopressin in the renal tubules has been demonstrated. Consequently, this may result in increased diuresis and dehydration of the body [20,21]. This is associated with increased water resorption from bile, thereby intensifying its concentration in the gallbladder. The formation of crystallisation nuclei is promoted by such a state [21]. The presence of microcrystals in turn promotes the formation of larger deposits of cholesterol, bilirubin, and calcium salts [21]. Small gallstones that have been formed can migrate through the common bile duct to the outlet with the pancreatic duct [19,21].

The passage, due to their hardness and irregular surface, can lead to damage to the biliary epithelium. Larger deposits are particularly dangerous because they cannot pass through the duodenal papilla [22]. The formed deposit will, therefore, remain in the anatomical space from which it cannot pass into the intestines. Furthermore, the process of crystallisation on its surface may be subject to further development until the formation of substantial crystals [23,24]. Alcohol has been shown to exert an unfavourable effect by increasing the tension of the sphincter of Oddi muscles [24]. Consequently, the release of pancreatic enzymes is impeded, resulting in their accumulation and premature activation. Postmortem observations also indicate the formation of protein deposits in the pancreatic ducts [24]. The dominant concentration is of lithostatin, classified as an albumin [25–27]. Its function is to inhibit the crystallisation of calcium carbonate in the pancreatic ducts [27]. Research conducted on the effect of alcohol in animal models provides evidence of increased lithostatin mRNA. Consequently, it has been demonstrated to increase production in pancreatic juice and may promote protein deposition in small ducts [28]. In the study by Brown et al. a multitude of disturbances in pancreatic tissue were observed in deceased patients. Researchers conducting macroscopic and histological evaluations of the organ observed edematous changes. In the context of the prevailing changes, the distinction was made between fat necrosis and the associated AP. The medical histories of patients frequently exhibit the presence of comorbidity. These include metabolic disorders, obesity, diabetes, and alcoholism (chronic alcohol consumption) [29].

## 3. Etiopathogenesis of AP

### 3.1. Oxidative stress

Under normal conditions, the cell achieves a state of redox homeostasis [30,31]. ROS perform many beneficial functions in cells. These molecules are produced and participate in the Krebs cycle, resulting in the formation of ATP. ROS also play a significant role in modulating the response to damaging factors. Nearly 90% of ROS is produced on the inner mitochondrial membrane [30]. Organelles such as the endoplasmic reticulum (ER) are also responsible for producing them. The formation of ROS is associated with the main function of ER’s primary function of post-translational protein modulation. Oxidative protein folding is the dominant process responsible for generating approximately 25% of ROS. Enzymes such as protein disulfide isomerase (PDI) and oxidoreductases facilitate the transfer of electrons to molecular oxygen [30]. Additional processes related to thiol bond reshuffling contribute to the generation of ROS by molecular oxygen. One mechanism that drives the formation of ROS is the release of calcium from the ER [31,32]. The intensification of release is associated with stress factors in the reticulum. Mitochondria capture Ca2+ ions through calcium channels. Higher concentrations of Ca^2+^ in the mitochondria promote improved oxygen metabolism and increased ATP production. However, production of intermediate products and ROS mediators also increases [30,32,33]. Once released from the mitochondria, they can interact with the ER. This results in the release of larger amounts of Ca^2+^ from the reticulum [30–32]. It is important to note that the oxidation and detoxification of toxic substances and drugs involves numerous metabolic pathways within cells. The detoxification process mainly occurs in hepatocytes, which are cells found in the liver. This process contributes to the depletion of the body’s antioxidant pool [30,34]. These processes can also occur in pancreatic cells in cases of excessive alcohol intake.

Therefore, alcohol is recognized as a leading cause of acute AP worldwide [34,35]. Metabolizing toxic compounds consumes important mitochondrial coenzymes, such as NAD (nicotinamide adenine dinucleotide) and NADP (nicotinamide adenine dinucleotide phosphate). This results in the accumulation of intermediate products of redox reactions. The group with the highest reactivity includes the hydroxyl radical (•OH), oxygen (O_2_), hydrogen peroxide (H_2_O_2_), and the superoxide anion (O_2_^–^) [31,33]. ROS are produced when chemical bonds are broken down by enzymes. These molecules have a free electron in their molecular structure [32]. This results in the high reactivity of ROS with organic compounds, including proteins, lipids, and nucleic acids. The inner mitochondrial structure is the first to be affected, including the cristae, mtDNA, and pore proteins. Consequently, ROS can more easily escape from the mitochondria into the cell cytoplasm [34,36]. Therefore, oxidative damage to cellular organelles, the cytoskeleton, or nuclear structures can lead to cell death. This process can occur through various mechanisms, including necrosis, apoptosis, necroptosis, and autophagy [36].

Hydrogen peroxide is an oxygen-containing free radical [37,38]. The concentration of H_2_O_2_ is a critical determinant of cell fate in pancreatic acinar cells [39]. Low concentrations (1–10 µM) preferentially promote the activation of the time- and concentration-dependent apoptotic cell death pathway, with minimal necrosis. The induction of apoptosis by ROS may be beneficial for eliminating compromised acinar cells without triggering inflammation-inducing necrosis. Higher concentrations (0.5–1 mM) induce rapid necrosis with minimal transient elevation of apoptotic cell death, exceeding twice the control value at two hours and remaining elevated throughout the experimental period [39]. This shift from apoptosis to necrosis is a key outcome of oxidative stress [38,39]. H_2_O_2_ also decreases the mitochondrial NADH/FAD redox ratio and mitochondrial membrane potential (ΔΨ_m) in a concentration-dependent manner, with maximal effects observed at 500 µM [39]. H_2_O_2_ also decreases the basal oxygen consumption rate (OCR) of acinar cells and causes concentration-dependent decreases in cellular ATP [39]. H_2_O_2_ at concentrations above 50 µM can inhibit plasmalemmal Ca^2+^-ATPase in pancreatic acinar cells, suggesting a role in excessive Ca^2+^ overload that drives necrosis [39,40]. Caspase-3 activity is differentially regulated by the extent of H_2_O_2_-induced ROS generation: lower levels increase activity, while higher concentrations are inhibitory [39]. Superoxide anion is an oxygen-containing free radical produced when molecular oxygen is reduced during normal cellular metabolism [38,41]. Superoxide dismutase (SOD) can spontaneously or enzymatically convert O_2_^-^ to H_2_O_2_. A loss of SOD activity increases oxidative stress-induced damage to DNA, lipids, and proteins [38,41].

Reactive nitrogen species (RNS) are also essential to the progression of AP and have destructive effects similar to those of ROS. RNS directly affect biomolecules, such as lipids and proteins, triggering pro-inflammatory signaling cascades that lead to immune responses. Progression of AP results in the induction of inducible nitric oxide synthase (iNOS) in tissues, producing a significant amount of highly reactive RNS, including nitric oxide (NO). While endogenous NO production is usually beneficial, uncontrolled and excessive NO production can cause tissue damage. Studies have shown that iNOS-deficient mice exhibit less severe pancreatic inflammation, suggesting a possible therapeutic function for iNOS inhibitors in AP [38]. Highly selective iNOS inhibitors have significantly improved experimental acute pancreatitis by effectively inhibiting iNOS activity [40].

### 3.2. Induction of the inflammatory response

Released DAMPs activate acinar cells, dendritic cells, and tissue macrophages. These molecules are ligands for a large group of pattern recognition receptors (PRRs), which include. Toll-like receptors (TLRs), C-type lectin receptors (CLRs), and NOD-like receptors (NLRs) [5]. Epithelial and glandular cells, including pancreatic acinar cells, contain TLRs, NLRs, P2X7 receptors, and receptors for advanced glycation end products (RAGE). These receptors, along with TNFR and IL-1R, are part of the canonical NF-κB pathway [42,43]. The most well-known receptor in AP is TLR, which forms a dimer when it binds to a ligand [43,44]. The next step is the phosphorylation of the intracellular domain, which mainly occurs on its tyrosine residues through various kinases. Then, a complex of MAL/MyD88 and TRIF (TIR-domain-containing adapter-inducing interferon-β) proteins forms and binds to specific intracellular receptors [44–46].

This stage involves kinases associated with Interleukin-1 Receptor-Associated Kinase 1 (IRAK) and TGF-β-activated kinases (TRAF and TAK). The TAK1 kinase undergoes phosphorylation due to the formation of the TRAF6 and TAB complex. Next, the cytosolic NF-κB heterodimer dissociates due to the action of IKK (inhibitor of nuclear factor kappa-B kinase). This results in the separation of the REL subfamily proteins (e.g. the p50 protein) from the inhibitory factor IκB [45–47].

### 3.3. Leukocyte response to cytokines

Local pancreatic damage activates tissue macrophages. It also promotes the migration of all leukocyte subpopulations. Two waves of white blood cell influx are observed during AP [48,49]. The first wave is associated with the release of DAMPs, cytokines (IL-8, IP-10, MCP-1, and RANTES), and oxidative stress. This leads to local extravasation and the migration of primarily neutrophils and monocytes [55,58]. This process occurs within an hour of the onset of an AP episode. The next stage involves an influx of adaptive immune response cells, including Th (CD4+) and Tc (CD8+) lymphocytes. These cells promote tissue regeneration and fibrosis after inflammation occurs [49,50].

#### 3.3.1. Neutrophils

Neutrophils express pattern recognition receptors (PRRs) on their surfaces. These receptors allow neutrophils to respond to signals, including those associated with cellular damage and pathogens [51]. This contributes to the multistep process of migrating to sites affected by cellular injury. The process begins with margination during passive flow through small capillaries. Engagement of surface receptors enables adhesion and rolling [52]. Contact with the endothelium and factors such as DAMPs also lead to initial activation [53–55].

This mechanism is evident in infectious processes and cellular injury. It is a critical factor in the pathogenesis of AP and its associated complications. In this context, granulocytes release large quantities of ROS and pro-inflammatory cytokines, such as IL-6, TNF-α, and IL-1β. Another effective mechanism that drives the inflammatory response is the release of neutrophil extracellular traps (NETs). NETs are networks of nuclear chromatin fibers encrusted with enzymes, such as myeloperoxidase (MPO) and cathepsin G [50,56,57]. This structure’s main function is to capture and neutralize pathogens. However, the chromatin released in this process also belongs to the group of DAMPs [48]. Studies on animal models have shown that NETs can increase damage at the site of infiltration by neutrophils. Furthermore, NET occlusions have been observed within the pancreatic ducts [50,58]. The release is triggered by the secretion of IL-8, IL-17a, and TNF. It is also triggered by the presence of microcrystals [59]. The most significant influence is exerted by IL-17a, which is mainly produced by a subpopulation of T helper lymphocytes (Th17) [60]. Interestingly, the number of Th17 lymphocytes in the pancreas is initially limited, resulting in a limited neutrophil response. In addition to activating neutrophils, Th17 lymphocytes influence the recruitment of macrophages [60].

#### 3.3.2. Macrophages and monocytes

In AP, besides neutrophils, monocytes and macrophages play a major role. Several types of macrophages are identified in an inflamed pancreas [50]. These include tissue macrophages, infiltrating monocytes, transitional macrophages, and monocytes. There are also two main subpopulations of macrophages: M1 and M2 [56,61,62]. As with other inflammatory diseases, the severity of inflammation depends on the number of recruited monocytes and their level of activation. These cells are stimulated by CC chemokines [56]. Important proteins from this subfamily include CCL2 (Monocyte Chemotactic Protein, MCP-1), CCL3 (Macrophage Inflammatory Protein, MIP-1α), CCL5 (RANTES), and CCL7 (MCP-3) [56,63,64]. During AP, pro-inflammatory cytokines activate macrophages in other tissues. The primary response is observed from macrophages in the lung alveoli, peritoneum, and liver [50]. Upon activation, Kupffer cells in the liver secrete numerous cytokines, including TNF-α, IL-6, and IL-1β [65]. These cells can also be a local source of reactive oxygen and nitrogen species, contributing to a systemic inflammatory response. Macrophages phagocytose DAMP molecules [31,66]. Phagocytes and dendritic cells are the main components of the antigen-presenting cell system. They present antigens to naïve T lymphocytes in the lymph nodes (LN) [67]. In the early stages, macrophages secrete pro-inflammatory cytokines, including IL-1β, IFN-γ, and TNF-α. These cytokines activate the pro-inflammatory M1 phenotype. Together with increased granulocyte recruitment, this induces a cytokine storm [62,68,69]. This phenomenon promotes the development of hyperactive leukocyte subpopulations in the bone marrow. Studies of animal models of severe AP demonstrate increased secretion of platelet-activating factor (PAF-RA) by neutrophils and macrophages [68].

#### 3.3.3. Lymphocytes

In the pathogenesis of AP, infiltration of the pancreas by adaptive immune response cells is observed. T cells are recruited into the bloodstream from the lymph nodes (LN) due to antigen presentation by APCs and direct interaction with DAMPs. The primary types of lymphocytes involved in this process are the CD4+ (T helper cells) and CD8+ (cytotoxic T cells) subpopulations [67,70]. In animal models, an influx of CD4+ and CD8+ lymphocytes is observed after the initiation of AP. Researchers observed the peak of organ damage and T lymphocyte activity eight hours after initiation [70]. These cells secrete numerous pro-inflammatory cytokines, including IFN-γ from Th1, IL-5 and IL-13 from Th2, and IL-17, IL-21, and IL-22 from Th17 [71,72]. CD8+ lymphocytes can also contribute to the intensification of inflammation. After infiltrating the inflamed tissue, these cells secrete cytotoxic proteins, including granzymes, perforins, TNF, and TRAIL [71,73,74]. Consequently, the apoptosis of damaged cells in the pancreatic parenchyma and acini is promoted [75].

#### 3.3.4. Platelets

Thrombocytopenia and an increased tendency toward thrombosis are associated with AP. These anucleate cells, the smallest in the bloodstream, are involved in hemostasis and the inflammatory response [76]. These processes require the following internal granules: α-granules, dense granules, and lysosomes [68]. α-Granules contain chemokines from the CXCL family (CXCL 1, CXCL 4, CXCL 5, CXCL 7, CXCL 12), CCL3 (MIP1a), and CCL5 (RANTES) [68,77,78]. They also contain coagulation factors, including TGF-β (transforming growth factor-beta), platelet-derived growth factor receptors (PDGF), and IL-1, IL-6, IL-10 [77–79]. Dense granules contain Ca^2+^, Mg^2+^, serotonin, and histamine. Lysosomes contain proteases, including cathepsin, acid phosphatase, and collagenase [80]. Platelets have PRRs. Thus, they play an auxiliary role in defending against pathogens and responding to DAMPs. Ligand binding to a PRR activates platelets, leading to their degranulation [61,68,78] Chemokines stored in platelet α-granules promote the recruitment of granulocytes and monocytes to inflamed pancreatic tissue. The release of interferons (α and β) and interleukins, particularly IL-6, influences megakaryocyte function, leading to a reduced production of platelets and change phenotype to hyperactive[68,73,76]. In addition, activated platelets could adhere to leukocytes in the inflammatory region. Furthermore, aggregates potentially amplifying local immune responses [76].

During the early phase of hospitalisation for acute pancreatitis, thrombocytopenia is a frequent finding. This decline in platelet count is likely multifactorial, reflecting both immune-mediated clearance of hyperactivated platelets and cytokine-driven suppression of megakaryopoiesis. Decrease of the inflammatory process leads to normalisation in platelets count. This corresponds to the period of discharge from the ward [81].

## 4. Biochemical markers and clinical scores for predicting the severity of acute pancreatitis

Although most patients with AP experience a mild disease course, around 20% develop complications, with mortality rates in complicated cases reaching up to 60%. Several approaches have been proposed for severity stratification in acute pancreatitis, including the use of single biochemical markers, imaging techniques, and complex scoring systems. These approaches are all aimed at facilitating the early detection of severe disease and optimising patient monitoring and management. Laboratory parameters provide an effective, rapid, effective, and cost-efficient way of predicting the risk of severe acute pancreatitis (SAP).

Of the single biochemical markers, C-reactive protein (CRP) is the most widely used and clinically valuable [82]. A recent meta-analysis including 41 studies involving 6156 patients confirmed the significant diagnostic value of CRP, with pooled sensitivities and specificities of 0.76 and 0.79, respectively, and an area under the curve (AUC) of 0.85 for predicting SAP [83]. These findings emphasise the reliability of CRP as an independent marker of disease severity, although its predictive accuracy is increased when combined with other biomarkers. Recent studies have indeed highlighted the diagnostic utility of procalcitonin (PCT), interleukin-6 (IL-6) and lactate dehydrogenase (LDH). Combined assessment of CRP, PCT, IL-6 and LDH achieves an area under the curve (AUC) of 0.98, indicating excellent diagnostic performance for the early identification of severe acute pancreatitis (SAP) and highlighting the potential of multimarker panels in clinical practice [82,84]. Furthermore Wang et al. suggested that a simpler combination of lipase, CRP and amylase could be used as an initial approach to risk stratification in patients AP [85].

These findings are consistent with those of other investigators who have reported that routine hematological and biochemical markers may predict AP-related complications. Specifically, patients with complications demonstrated elevated levels of white blood cells (WBC), neutrophils, CRP, and erythrocyte sedimentation rate (ESR), along with decreased red blood cell count and hemoglobin (Hb) concentration. Moreover ESR, platelet count, and Hb may be an effective tool for identifying pseudocysts in AP patients [86]. Chen et al. analysed thirty clinical and laboratory variables, fifteen of which were identified as significant predictors of AP progression: (WBC, fibrinogen (FIB), alanine aminotransferase (ALT), alkaline phosphatase (ALP), ALP-to-hemoglobin ratio (ALP/Hb), LDH, LDH-to-hemoglobin ratio (LDH/Hb), urea, creatinine, CRP, PCT, blood glucose, and the Acute Physiology and Chronic Health Evaluation – II (APACHE-II), Bedside Index for Severity in AP (BISAP), and Sequential Organ Failure Assessment scores (SOFA). Among these, six parameters demonstrated the strongest association with SAP: ALP, ALP/Hb, LDH, LDH/Hb, CRP, and blood glucose concentration. Notably, the ALP/Hb and LDH/Hb ratios exhibited the highest predictive value for the development of SAP [86]. Dronov et al. demonstrated that the BISAP score, sFGL2, CRP, D-dimer, and intra-abdominal pressure may contribute to the prediction of clinical deterioration of acute pancreatitis. Moreover, elevated levels of sFGL2, d-dimer, and intra-abdominal pressure were identified as predictors of progressive necrotic changes in pancreatic and retroperitoneal tissue [87]. Beyond biochemical and clinical approaches, novel biomarkers such as serum trypsinogen-2 have proven useful in the early diagnosis of post- endoscopic retrograde cholangiopancreatography (ERCP) pancreatitis. Levels declining between 1 and 2 h post-procedure reliably exclude disease progression [88].

The Early Achievable Severity Index (EASY) score allows patients at high risk of SAP to be identified early on in their hospital stay. This is achieved by using six routinely available clinical parameters (respiratory rate, body temperature, abdominal muscular reflex, gender, age, and glucose level). The model, which has been validated in large international cohorts, demonstrated high predictive accuracy (AUC 0.81) and is accessible through a free web application [89]. Similarly, Vannier et al. proposed the Admission Severe Acute Pancreatitis (ASAP) score, a simple tool derived from parameters at the time of admission. Incorporating hypothermia, reduced oxygen saturation, hypoalbuminemia, and elevated creatinine, the ASAP score achieved strong predictive accuracy (AUC 0.82), outperforming established systems such as SOFA, persistent SIRS (systemic inflammatory response syndrome). Its performance was subsequently validated in an independent cohort, further supporting its clinical utility for early risk stratification [90]. In addition, a prediction model for infected necrotizing pancreatitis has recently been proposed to address the need for the early identification of patients requiring interventional therapy. Developed from a retrospective cohort of 705 patients, the model incorporated routine laboratory and clinical variables. CRP, albumin, creatinine and alcoholic etiology were identified as independent predictors. With an AUC of 0.82, the model outperformed single biomarkers and the APACHE II score, demonstrating its potential to guide the timely management of necrotizing pancreatitis [91].

Advances in deep learning applied to imaging have shown promise in pancreatitis diagnosis and severity assessment. A novel CT-based diagnostic model demonstrated excellent efficacy, achieving an AUC of 0.993 in the test set and 0.850 in external validation. The model also provided reliable lesion segmentation, highlighting the increasing importance of artificial intelligence in evaluating AP [92].

To summarize the above, although there are many scoring systems and associated clinical and laboratory tests used to assess the severity and prognosis of AP, it is still unclear which of them is the most effective and should be used first; the topic is important and requires further research and development.

## 5. Complications of AP

### 5.1. Necrosis and bacterial superinfection

According to the revised 2012 Atlanta Congress guidelines, the onset of an AP episode is considered to have begun when patient experiences pancreatic pain [6]. The longer it takes to diagnose the condition and implement optimal therapy, the worse the patient’s prognosis becomes [6]. Clinical observations suggest that the time between the first two days are crucial for progression of AP. In the initial period, about 30% of patients may develop a severe form of inflammation that leads to necrotizing pancreatitis [75,93]. Imaging studies allow the observation of necrotic changes in the pancreas and surrounding adipose tissue to be observed in patients [75]. From the initial period of necrosis onwards, the immune system may become intensely activated. Prolonged necrotic areas can lead to superinfection with the intestinal flora (Figure 2) [94]. This condition most often develops due to immunosuppression caused by the compensatory anti-inflammatory response syndrome [95]. These processes are a mechanism maintaining the balance of inflammatory processes in the body.

They are accompanied by an increased secretion of IL-1ra and IL-10, leading to reduced leukocyte antigen expression by monocytes [96,97]. Changes in the number and proportions of individual T lymphocyte populations are also possible [98]. Endotoxins contained in the walls of Gram-negative bacteria influence this phenomenon [99]. They lead to decreased IL-2 secretion, which contributes to the weakening of T and NK lymphocyte proliferation [99,100]. Factors such as necrosis, mechanical injury or the severity of the primary disease promote the occurrence of SIRS [101]. Persistent inflammation promotes the development of distant organ failure. Sometimes patients develop lung failure due to severe AP [102,103]. It is caused by the activation of alveolar macrophages by circulating pro-inflammatory cytokines. The worst prognosis in AP is multi-organ failure, which can lead to death [101–103].

### 5.2. Pancreatic fibrosis

Following the inflammatory response, the removal of cellular debris is essential for the regeneration process. These structures are detected by a large family of scavenger receptors (SRs). SRs are present on the surface of phagocytic cells, including macrophages. Binding of ligands to the receptor activates phagocytosis and numerous intracellular pathways, leading to the secretion of cytokines and cellular growth factors [35]. They exhibit a significant potential to modulate the immune response and tissue regeneration processes [62]. The secreted TGF-β1 and PDGF activate fibroblasts to proliferate. These fibroblasts then transform into myofibroblasts. This process involves the autophosphorylation of the STAT3 kinase pathway (transcription activator) and JAK 1/2 (Janus kinase) pathways. Consequently, the transcription of collagen fibre genes is activated [62,104,105]. The next stage of the process is the secretion and accumulation of collagen in the extracellular matrix (ECM). This condition is clinically referred to as pancreatic fibrosis. However, alcohol abuse can directly activate the transformation of fibroblasts [104,106,107].

This is referred to as alcoholic pancreatic fibrosis. Episodes of AP can be observed in such cases. These are caused by structural and patency disorders of the pancreatic ducts. Interestingly, myofibroblasts can also modulate the normal structure of the ECM. They have the ability to produce enzymes from the metalloproteinase group that degrade the ECM. Myofibroblasts can also undergo re-transformation into fibroblasts [33,104,107].

Pancreatic stellate cells (PSCs) are a distinct population of mesenchymal cells found in the pancreas. They make up around 4–7% of the total parenchymal cell population. Considerable advances have been made in defining their physiological functions and pathological relevance since their initial description nearly three decades ago [108]. PSCs belong to the wider group of retinoid-storing stellate cells found in various organs and exhibit close phenotypic and functional similarities to hepatic stellate cells (HSCs). Transcriptomic and proteomic analyses have revealed significant similarities between HSCs and PSCs, while also emphasising organ-specific adaptations that reflect the pancreatic microenvironment [108,109]. HSCs are well established as being of mesenchymal origin, and current evidence indicates that PSCs likely share a similar developmental lineage [108].

In a healthy pancreas, PSCs exist in a quiescent state in which they store fat. They are predominantly located in the periacinar and interlobular regions, where they are characterised by cytoplasmic lipid droplets that are rich in retinoids [108,109]. Under physiological conditions, PSCs contribute to the maintenance of normal ECM homeostasis by regulating the synthesis and degradation of ECM proteins. They have also been proposed to act as intermediary cells in cholecystokinin-mediated regulation of exocrine secretion. In addition to these structural and regulatory functions, quiescent PSCs exhibit immunological activity, including recognition of pathogen-associated molecular patterns (PAMPs) *via* Toll-like receptors, as well as the ability to phagocytose necrotic acinar cells and infiltrating neutrophils. Furthermore, PSCs express stem cell-associated markers such as nestin, CD133, SRY-box9, and growth differentiation factor 3, which suggests that they may have a role as pancreatic progenitor cells [108].

Activation of PSCs represents a central event in the development of pancreatic fibrosis, a hallmark of chronic pancreatitis and pancreatic cancer [108,110]. During this process, PSCs undergo a phenotypic transition from their quiescent, vitamin A-containing state to a myofibroblast-like state [108,111]. Activated PSCs are defined by the loss of cytoplasmic vitamin A lipid droplets and the de novo expression of α-smooth muscle actin (α-SMA), which is the most widely recognized marker of their activated state [108,111,112]. Functionally, these cells display enhanced proliferative and migratory capacity, increased synthesis and deposition of ECM proteins such as collagen types I and III and fibronectin, and the production of matrix metalloproteinases (MMPs) together with their tissue inhibitors (TIMPs), thereby contributing to ECM remodeling [108,112,113]. In addition, activated PSCs secrete a broad array of growth factors, including PDGF, fibroblast growth factor (FGF), TGF-β1, and connective tissue growth factor (CTGF), as well as pro-inflammatory cytokines and chemokines such as interleukin (IL)-1β, IL-6, IL-8, RANTES, tumor necrosis factor-α (TNF-α), and monocyte chemoattractant protein-1 (MCP-1) [108–110]. Collectively, these features endow PSCs with a high mitotic index, strong motility, and a central role in driving both fibrogenesis and inflammation within the diseased pancreas [108,112,113].

The initiation of AP is largely attributed to toxic calcium (Ca^2+^) signaling within acinar cells [110,114]. That lead to the intracellular trypsinogen activation, ATP depletion, and necrotic cell death. Alcohol-derived fatty acid ethyl esters (FAEEs), bile acids, and mechanical or chemical insults have each been shown to induce sustained intracellular Ca^2+^ elevations, primarily through the opening of calcium release-activated calcium (CRAC) channels. These pathological Ca^2+^ signals drive not only acinar cell necrosis, but also contribute to the activation of surrounding stromal and immune cells [114].

The inflammatory cascade that follows acinar cell injury is amplified by [114]. Upon activation, PSCs secrete pro-inflammatory mediators, thereby promoting leukocyte recruitment and transmigration into injured tissue [108–110]. Calcium signaling also regulates PSC activity directly: bradykinin, bile acids, FAEEs, and even the SARS-CoV-2 spike protein have been shown to elicit robust Ca^2+^ transients in stellate cells, triggering NO production and cytokine release [114]. Through reciprocal interactions with macrophages and other immune cells, PSCs thus serve as amplifiers of the inflammatory response in AP [109].

### 5.3. Mechanisms promoting progression to neoplastic changes

Acute pancreatitis (AP) can lead to both short- and long-term complications (Figure 2).

The microenvironment of areas affected by inflammatory and necrotic changes favors tumor development [115]. This process is highly complex, and some of the mechanisms are not yet fully understood. It has been suggested that the increased risk of cancer is due to numerous tissue injuries during intense disease processes (Table 2) [115–117]. Metaplastic cells can be observed in inflamed regions [118]. An additional factor that drives carcinogenesis is the occurrence of mutations in the KRAS (Kirsten rat sarcoma viral oncogene) oncogene [118,119]. The combination of epithelial damage, ECM modulation, and mutated KRAS promotes neoplasia [118]. Alonso-Curbelo et al. observed chromatin dysregulation in pancreatitis as early as 48 h after injury. This process involves disrupting of normal regenerative processes and transitioning to neoplastic transformation pathways [120].

**Table 2.** Probability of long-term complications of acute pancreatitis [121–123].

Author, n cases	Country; year of publication	Subgroup analyses	OR/HR	Conclusion
Sadr-Azodi et al. AP 49 749 C: 138 750	Sweden; 2018	Gallstone-related pancreatitis		In the study, the causes of AP were categorized, and the risk of pancreatic cancer (PC) was analyzed. Gallstone-related pancreatitis increases the risk of PC only in the 2–3 and 3–4 year periods following pancreatitis. However, non-gallstone related AP leads to an increased risk of PC in all studied periods. The highest hazard ratio (HR) values were observed 2–3 years after pancreatitis
		2–3 years	HR 2.45 (95% CI 1.00–6.00	
		3–4 years	HR 3.30 (95% CI 1.21–5.03)	
		4–5 years	HR 0.76 (95% CI 0.17–3.34)	
		Non-gallstone related acute pancreatitis		
		2–3 years	HR 12.96 (95% CI 5.43–30.94)	
		3–4 years	HR 2.54 (95% CI 1.22–5.26)	
		4–5 years	HR 4.88 (95% CI 2.23–10.71)	
Kirkegård J et al. AP 49 749 C: 208 340	Denmar; 2018	>2 to 5 years post pancreatitis	aHR 2.43; 95% CI 1.73–3.41	Patients with AP were included in the study. An increased risk of PC of 2.43 times was observed in the period 2–5 years after the onset of AP. This risk decreased after 5 years, but remained 2.02 times higher than in the healthy control group.
		> 5 years post pancreatitis	aHR 2.02; 95% CI 1.57–2.61	
Park BK et al. AP 25 488 C: 138.750	Korea; 2023	2-4 years post pancreatitis	3.62 (95% CI 2.26–4.91)	During the study period, which lasted around 5.4 years on average PC was observed in 1.9% of patients. In the control group, this figure was only 0.2%.

#### 5.3.1. Immune response cells’ role

They perform various functions in the inflamed tissue. In the context of complications, the most important tasks are the production of cytokines and cellular growth factors [118]. In tumour development, a significant proportion of cells are fibroblasts, macrophages, neutrophils, and platelets. Tumor-associated macrophages (TAM) are divided into two subpopulations. The first is the M1 subpopulation, which exhibits a highly pro-inflammatory character. This subpopulation produces IL-1, −6, −8, −12, TNF-α, and ROS, RNI [61,77]. They influence the activation of lymphocytes, including Th1 cells. The secretion of ROS in combination with Tc cells present will have a cytotoxic effect on tumour cells and cells damaged by inflammation. In contrast, the M2 macrophage subpopulation exhibits an immunosuppressive effect. This effect occurs through the secretion of IL-4, −13, and IL-12, −23. This leads to a reduction in T lymphocyte response. This may result in a weakened anti-tumor response. TAM-secreted angiogenesis and lymphangiogenesis stimulators (VEGF, TNF-α, and IL-8) exhibit a pro-tumour character [61].

### 5.4. Interstitial edematous pancreatitis

Upon its occurrence, low-grade pancreatic tissue damage is noted. Clinical data generally confirm the absence of necrotic changes and the self-limiting nature of the edema [108]. Throughout the course of interstitial edematous pancreatitis, an increase in the concentration or activity of pancreatic enzymes in the serum is observed. On CT, enhancement of the pancreatic parenchyma without characteristic necrotic features is seen [124,125].

Another possible complication is a pancreatic pseudocyst. This condition involves the formation of well-defined fluid collections containing inflammatory fluid. Such changes usually do not include necrotic foci, although they may be present to a limited extent. The fluid is pancreatic juice, which is produced when the main pancreatic duct or its side branches close or rupture. The next stage is the leakage of this fluid, which forms pseudocysts [126]. These changes tend to reduce over time, and sometimes surgical drainage of the cysts is required [126–128].

## 6. Summary

Acute pancreatitis (AP) is an inflammatory disorder that is becoming more prevalent. It is characterized by premature enzyme activation, oxidative stress, and dysregulated immune responses. These factors drive pancreatic injury, systemic complications, and progression to fibrosis and malignancy. Several approaches have been developed to address the clinical heterogeneity of AP, including biochemical markers, hematological indices, and clinical scoring systems. More recently, deep learning-based imaging tools have been developed for severity prediction. However, significant challenges remain in determining the most reliable prognostic methods and identifying biomarkers that can accurately predict clinical deterioration and long-term outcomes. Future research should focus on validating predictive models, discovering more specific biomarkers, and optimizing clinical strategies to reduce disease burden. These advances are critical for improving early risk stratification, guiding targeted interventions, and ultimately enhancing patient outcomes in AP.

## Data Availability

No new data was generated during the study.

## Abbreviations

AP: Acute pancreatitis
ALP: Alkaline phosphatase
ALP/Hb: ALP-to-hemoglobin ratio
ALT: Alanine aminotransferase
APACHE-II: Acute physiology and chronic health evaluation-II
ASAP: Admission severe acute pancreatitis
BISAP: Bedside index for severity in AP
CARS: Compensatory anti-inflammatory response syndrome
CECT: Contrast-enhanced computed tomography
CLR: C-type lectin receptors
CRAC: Calcium release-activated calcium
CREA: Creatinine
CRP: C-reactive protein
CTGF: Connective tissue growth factor
DAMP: Damage-associated molecular patterns
EASY: Early achievable severity index
ECM: Extracellular matrix
ER: Endoplasmic reticulum
ERCP: Post-endoscopic retrograde cholangiopancreatograph
ESR: Erythrocyte sedimentation rate
FAEE: Alcohol-derived fatty acid ethyl ester
FGF: Fibroblast growth factor
FIB: Fibrinogen
H_2_O_2_: Hydrogen peroxide
HMGB1: High mobility group 1 protein
HO•: Hydroxyl radical
HSP: Heat shock proteins
iNOS: Inducible nitric oxide synthase
IRAK: Interleukin-1 receptor-associated kinase 1
JAK: Janus kinase
KRAS: Kirsten rat sarcoma virus
LDH: Lactate dehydrogenase
LDH/Hb: LDH-to-hemoglobin ratio
LN: Lymph node
MMP: Matrix metalloproteinase
MRI: Magnetic resonance imaging
NAD: Nicotinamide adenine dinucleotide
NADP: Nicotinamide adenine dinucleotide phosphate
NET: Neutrophil extracellular traps
NLR: NOD-like receptors
NO: Nitric oxide
O2: Oxygen
O2•: Superoxide anion
OCR: Oxygen consumption rate
PAF-RA: Platelet-activating factor
PAMP: Pathogen-associated molecular patterns
PCT: Procalcitonin
PDGF: Platelet-derived growth factor receptors
PDI: Protein disulfide isomerase
PRR: Pattern recognition receptors
PSC: Pancreatic stellate cell
RAGE: Receptor for advanced glycation end-products
RNS: Reactive nitrogen species
ROS: Reactive oxygen species
SAP: Severe acute pancreatitis
SDI: Socio-demographic index
SIRS: Systemic inflammatory response syndrome
SOD: Superoxide dismutase
SOFA: Sequential organ failure assessment scores
SR: Scavenger receptor
TGF-β: Transforming growth factor-β
TIR: TIR-domain-containing adapter-inducing interferon-β
TLR: Toll-like receptors
USG: Ultrasound
α-SMA: α-Smooth muscle actin
